# Intrathecal Amylin and Salmon Calcitonin Affect Formalin Induced c-Fos Expression in the Spinal Cord of Rats

**Published:** 2014-11

**Authors:** Zahra Khoshdel, Mohammad Ali Takhshid, Ali Akbar Owji

**Affiliations:** 1 Department of Biochemistry Recombinant Protein Laboratory, School of Medicine, Shiraz University of Medical Sciences, Shiraz, Iran;; 2 Diagnostic Laboratory Sciences and Technology Research Center, School of Paramedical Sciences, Shiraz University of Medical Sciences, Shiraz, Iran;; 3 Research Center for Psychiatry and Behavioral Sciences, Department of Psychiatry, Hafez Hospital, Shiraz University of Medical Sciences, Shiraz, Iran

**Keywords:** Rats, Spinal cord, Proto-oncogene proteins c-fos, Salmon Calcitonin

## Abstract

**Background:** Amylin and Salmon Calcitonin belong to the calcitonin family of peptides and have high affinity binding sites in the rat spinal cord. The aim of this study was to characterize receptors for Amylin and Salmon Calcitonin functionally in the spinal cord of rats. We assessed the expression of c-Fos in response to intraplantar formalin in the lumbar regions of the spinal cord in conscious rats.

**Methods:** Amylin (0.05 nmoles) or Salmon Calcitonin (0.005 nmoles) was administered intrathecally (i.t.) 10 minutes before the start of the formalin test. Antagonists were injected intrathecally 10 minutes before the administration of either of the peptides.

**Results:** Two hours after formalin stimulation, rats pretreated intrathecally by either Amylin or Salmon Calcitonin, showed lower numbers of c-Fos immunoreactive nuclei in their lumbar spinal cord as compared to rats pretreated with saline. These effects were reversed upon co-administration of either of the Amylin antagonists AC187 or rat amylin_8-37_, but not rat α-CGRP_8-37_^. ^A few cells with c-Fos immunoreactivity were found in the lumbar spinal cord of rats two hours after i.t. injection of saline, Amylin and/or Salmon Calcitonin. However, Fos-like immunoreactivity was increased in the lumbar spinal cord two hours after i.t. treatment of either of the antagonists AC187 and rat amylin_8-37_,when compared to saline treated rats.

**Conclusion:** Both Amylin and Salmon Calcitonin inhibit formalin induced c-Fos expression in the rat lumbar spinal cord when administered intrathecally. Effects of the two peptides were possibly produced by undefined receptors.

## Introduction

Amylin (AMY) and salmon calcitonin (sCT) have structural similarities and belong to the calcitonin family of peptides. This family also includes calcitonin, two distinct forms of calcitonin gene-related peptide (α-CGRP and β-CGRP), adrenomedullin and AM2, also known as intermedin.^1^^,^^2^ These peptides are involved in a wide variety of biological functions and hence their receptors have therapeutic implications in many disease states.^3^^-^^5^ The pancreatic hormone, AMY, works with insulin in glucose regulation and energy balance.^6^ Actions of AMY in the CNS include regulation of appetite and adiposity.^7^ Although controversial, AMY has also been reported to play a role in nociception.^8^^,^^9^

AMY has not been reported to be synthesized in the brain, but AMY-like immunoreactivity is shown in the spinal cord.^9^^,^^10^ The Dorsal Root Ganglion (DRG) is the nervous tissue that shows both mRNA^11^^-^^13^ and immunoreactivity^9^^,^^13^ for AMY in rats. AMY has also been reported to pass through the blood brain barrier.^14^ Initially, CNS binding sites for radiolabeled forms of AMY^15^ and sCT^16^ were reported in brain areas as the nucleus accumbens, area postrema, dorsal raphe and subfornical organ. However, despite binding sites for sCT is shown in the spinal cord of rats,^16^ but AMY binding sites in this tissue have not been characterized. Later, it was shown that AMY activates receptors composed of a splice variant of the calcitonin receptor [CTR (a)/CTR (b)] and one of the receptor activity-modifying proteins (RAMPs). Thus, CTR dimerizes with either of the RAMPs 1-3 to form receptors referred to as AMY1, AMY2 and AMY3. Subscript ‘a’ or ‘b’ define which splice variant of the CTR is in the complex.^2^ CTR does not require RAMP to bind and respond to calcitonin. It is noteworthy that although mRNAs encoding the three RAMPs are expressed in the rat spinal cord,^17^^,^^18^ but the expression of CTR encoding mRNA is not clearly shown in this tissue. Therefore, the molecular identity of AMY action sites in the spinal cord remains to be established.

N-terminally truncated forms of CT-family of peptides, act as antagonists.^19^ rAMY_8-37_ has been reported to antagonize various responses to AMY with negligible potencies or affinities,^20^^,^^21^ despite reports of effective antagonism.^22^^,^^23^ However, AC187 is a potent and selective AMY antagonist^24^ that has been shown to discriminate between CGRP and AMY receptors in transfected cells.^21^

The association of another G protein-coupled receptors (GPCRs), the calcitonin receptor-like receptor (CLR), and RAMP1 generates the α-CGRP_8–37_ sensitive CGRP1 receptor.^25^^-^^27^ AMY and sCT are shown to bind CGRP1 receptors both in native tissues and in cellular systems.^2^ α-CGRP_8-37_ is reported to be an antagonist at AMY receptors as well.^28^^,^^29^ It is well established that CGRP participate in nociceptive transmission.^30^

The expression of immediate-early genes such as c-Fos is a marker of neuronal activity. We have previously reported that CGRP is stimulatory to spinal neurons in terms of increased cAMP accumulation^31^ and induced c-Fos expression.^32^^,^^33^ Here, we sought to verify the effects of AMY and sCT on the expression of the c-Fos in the spinal cord of rats. Further, we used AMY antagonists to discriminate between the CGRP and AMY receptors that mediate any possible effects of AMY and sCT on the c-Fos expression. Hind paw injection of formalin produces peripheral inflammatory responses that lead to increased c-Fos expression in the lumbar spinal cord. We also deployed this method to investigate the effects of AMY and sCT on the formalin induced c-Fos expression in the spinal cord of rats.

## Materials and Methods

*Animal Treatments and Surgery*

Forty-five male Sprague-Dawley rats bred at Shiraz University of Medical Sciences and weighing 250±20g were randomly divided into 15 groups. Animals were anesthetized with ketamine (50 mg/kg)+xylazine (5 mg/kg) and intrathecal catheterization was performed as described by Yaksh and Rudy.^34^ Briefly, a polyethylene catheter (PE-10, Betcton Dickenson, San Jose, CA) was stretched in a hot water bath at 72ºC to reduce its diameter, and 7.5 cm length of the elongated part of the catheter was threaded caudally into the subarachnoid space through a slit in the atlanto-occipital membrane. The rostral part was sutured to the adjacent muscles to immobilize the catheter and the wound was closed in two layers with 4-0 silk. The position of the caudal tip was always confirmed after the animals were sacrificed. Rats showing neurological deficits during the recovery period of seven days were excluded from the study. The Medical and Research Ethics Committee of the Shiraz University of Medical Sciences approved all experimental protocols.

*Chemicals*

Rat AMY and other peptides were obtained from Bachem Americas, Inc. (Torrance, CA.). The peptides were dissolved in sterile saline such that the final doses delivered (in 10 µl of vehicle) were as follows; AMY 0.05 nmoles, sCT 0.005 nmoles, rAMY_8-37_ 1.00 and 2.50 nmoles, acetyl-(Asn30, Tyr32)-calcitonin_8-32 _(AC187) 1.00 and 2.50 nmoles and rat α-CGRP_8-37 _2.50 nmoles. Peptides and antagonist concentrations were determined in pilot experiments (data not shown).

Formalin (37%), sucrose (analytical grade), glycerol (85-88%), paraformaldehyde, H_2_O_2_ (30%) were obtained from Merck Company. Triton-X100 (laboratory grade) was purchased from Sigma-Aldrich. DAB (diaminobenzidine) was purchased from Dako North America Inc.

*Injections and Tissue Preparation*

Peptide agonists and antagonists were administered i.t. in volumes of 10 µl, followed by 10 µl flushes of normal saline to clear the catheter. Antagonists were administrated 10 minutes prior to the injection of AMY and or sCT. Formalin (2.5%, 50 uL) or saline was injected into the hind paw of rats 15 min after they received i.t. injection of the drug or saline. Control rats were treated i.t. with 10 µl of saline prior to an injection of 50 µl of saline into one hind paw. Two hours after intrathecal injections, rats were deeply anesthetized and were perfused through the heart with 200 ml saline followed by 500 ml ice-cold 4% paraformaldehyde in 0.l M phosphate buffer. The L3-L5 spinal segments were removed and postfixed for 2 h and then transferred into the cryoprotection solution containing 30% sucrose in 0.1 M phosphate buffered saline (PBS) overnight at 4ºC. Frozen serial sections (40 µm) were cut in the transverse plane using a cryostat device. Every third section (80 µm intervals) was collected as free-floating sections and maintained at -25°C until immunohistochemical analysis. 

*Immunohistochemistry and Counting of c-Fos Protein Immunoreactive Nuclei*

Immunohistochemistry was performed by a horseradish peroxydase (HRP) method^37^ with a polyclonal antibody against c-Fos (Santa Cruz Biotechnology, Santa Cruz, CA). Briefly, free-floating spinal sections were washed with PBST and transferred into 0.3% H_2_O_2_ in PBST for 15 minutes to inhibit endogenous peroxidases. After blocking with 3% normal goat serum, sections were incubated in primary polyclonal anti-c-Fos antibody (diluted 1:300) for 24 h at 4°C. Visualization of the antigen-antibody complex was performed by using ready-to-use goat anti mouse EnVision-HRP enzyme conjugate for 40 minutes (Dako, Trappes, France). The peroxidase activity was visualized with 0.05% diaminobenzidine (DAB) and 0.3% hydrogen peroxide. The primary antibody was omitted in the case of immunohistochemical negative control sections. Sections were then rinsed with PBS, mounted onto slides with glycerol and cover slipped. Sections were examined by light field microscopy at 10× magnification to localize c-Fos labeled nuclei. Only those cells with stained round nuclei identified at 10× objective lens were counted. Following peripheral noxious stimuli, spinal neurons that express c-Fos were located in laminae I and II, and laminae V and VI of the dorsal horn.^35^ Therefore, we identified gray matter distribution of c-Fos nuclei in the laminae I- II of the superficial dorsal horn and laminae IV-VI of the neck of the dorsal horn. This was carried out in accordance with the cytoarchitectonic organization of the spinal cord.^36^ For each animal, 8–10 sections of the lumbar (L3-L5) spinal cord were examined. The average number of c-Fos positive nuclei in the three defined regions was calculated by averaging the counts made in 8–10 sections per spinal cord from three rats in each treatment group and expressed as the mean±SEM.

*Statistical Analysis*

All results are expressed, as mean±SEM. Analysis of variance (ANOVA) was conducted using computer software (SPSS Inc, USA) for comparison across the experimental conditions, considering the number of spinal c-Fos positive nuclei in the laminar regions throughout the lumbar segment of the spinal cord. The Tukey test was used for post-hoc analysis. Differences were considered to be statistically significant if P<0.05. One-way ANOVA was performed after normal distribution of data was verified by the Shapiro-Wilk test. 

## Results

*Intrathecal* *Administration of Saline, AMY and/or sCT*

**Figure 1:**
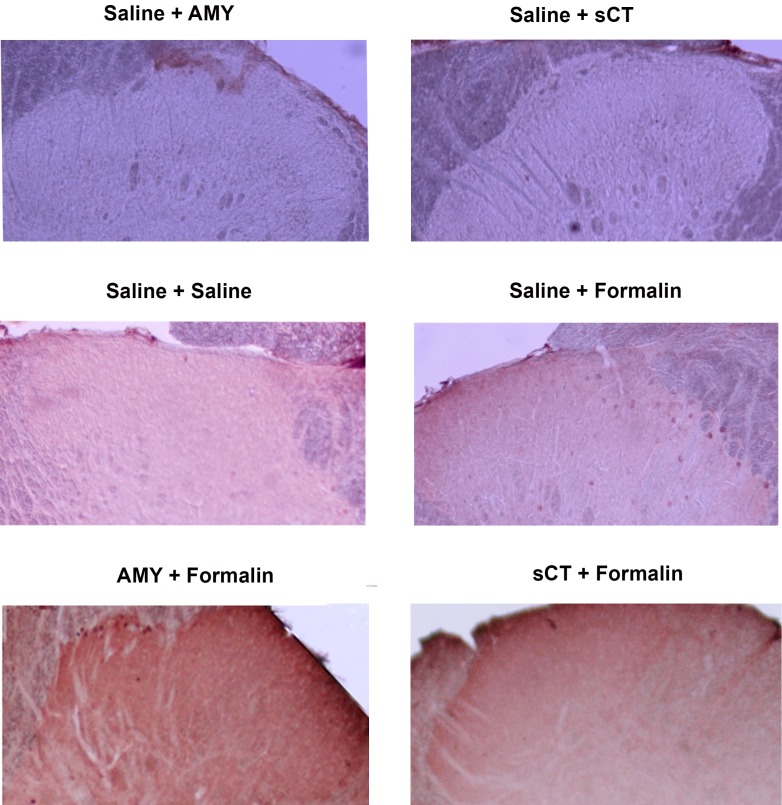
Photomicrographs of Fos-like immunoreactive neurons in the superficial (laminae I-II) dorsal horn of the lumbar (L3-L5) spinal cord. Rats were treated i.t. by saline and/or peptides prior to the injection of formalin into the hind paw. Pictures were taken using a 10× objective.

As shown in figure 1, control rats showed a few c-Fos positive nuclei scattered throughout the dorsal horn without any clustering or apparent pattern, 2 hours after they received intrathecal saline. Thus, nonspecific effects produced by i.t. saline, mechanical perturbations of the spinal cord by intrathecal catheter or injection of normal saline into one hind paw were not able to nonspecifically evoke the expression of Fos-like immunoreactivity. Intrathecal injection of either AMY or sCT failed to cause any significant increases in the c-Fos expression in the lumbar spinal cord of rats. In all animals, a few labeled cells were scattered throughout the dorsal horn without any clustering or apparent pattern. Data are shown in figure 1, with the number of c-Fos positive nuclei being summarized in table 1.

**Table 1:** Effects of AMY related peptides on the c-Fos expression induced by intraplantar formalin at the L3-L5 levels of the rat spinal cord

**Treatments (i.t.)**	**Total c-Fos Immunoreactive nuclei in laminae I-II and IV-VI **
Control†	5.54±0.79a
Saline	38.97±4.35d
AMY	14.28±1.19a,b
sCT	4.00±1.15a
CGRP_8-37_	26.83±2.48c,d
AMY+rAM_8-37 _(1 nmoles/10uL)	27.33±1.45c
AMY+AC187 (1 nmoles/10uL)	18.66±1.76b,c
(2.5 nmoles/10uL)	28.33±2.60c,d
sCT+rAM_8-37 _(1 nmoles/10uL)	18.66±1.76b,c
(2.5 nmoles/10uL)	19.00±3.50b,c
sCT+AC187 (1 nmoles/10uL)	8.06±2.40a
(2.5 nmoles/10uL)	29.66±2.02c,d
sCT+CGRP_8-37 _(2.5 nmoles/10uL)	6.16±2.94a,b
AMY+CGRP_8-37 _(2.5 nmoles/10uL)	11.73±1.88a,b

Data are Mean±SEM (n=3) of the number of c-Fos positive nuclei per section. One-way ANOVA showed significant differences among the groups (F (10, 22)=35.24, P=0.00). Data with superscript letters a,b,c and d differ from each other by P=0.05 or less. Peptides, AMY (0.05 nmoles/10uL) and /or sCT (0.005 nmoles/10uL), were injected i.t. before formalin administration into the hind paw. Antagonists were injected i.t. prior to the peptides. †Saline was injected in the plantar surface instead of formalin.

*Effects of i.t. Pretreatment with AMY and sCT on the Formalin-Induced c-Fos Immunoreactivity in the Spinal Dorsal Horn*

Formalin, when injected in the hind paw, is known to increase c-Fos expression in the spinal dorsal horn ipsilateral to the injection site. As shown in figure 1, two hours after hind paw injection of formalin, the number of c-Fos positive nuclei was increased on one side of spinal dorsal horn of rats pretreated with i.t. saline. However, both AMY (0.05 nmoles) and sCT (0.005 nmoles) decreased the number of c-Fos-positive neurons in the dorsal horn of the spinal cord when injected intrathecally 15 minutes before formalin injection (table 1). These effects of AMY and sCT were statistically significant (P=0.00 and P=0.00 respectively) when compared with the corresponding parts in the spinal cord of rats treated i.t. with saline prior to formalin injection.

*Effects of Antagonists of AMY and sCT on the Formalin-Induced c-Fos Expression in the Spinal Cord*

**Figure 2:**
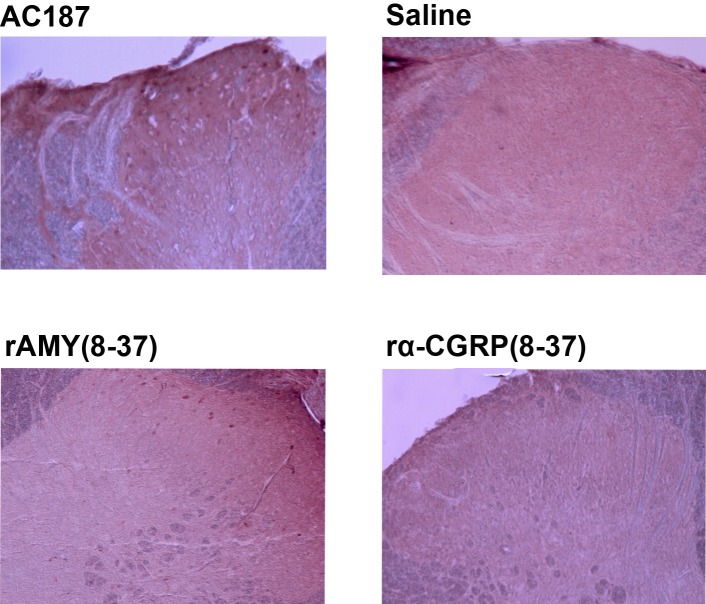
Fos-like immunoreactive neurons in the superficial (laminae I-II) dorsal horn of the lumbar (L4-L5) spinal cord. Saline and/or antagonist peptides were administered i.t. prior to the injection of saline into the hind paw. Pictures were taken using a 10× objective.

Table 1 presents the summarized data. The decreasing effects of i.t. AMY (0.05 nmoles) on c-Fos expression in the dorsal spinal neurons were antagonized when animals were pretreated i.t. (1 nmoles/10uL) by either AC187 or rAMY_8-37_. A statistical analysis by Tukey test revealed that this effect was significant in the case of rAMY_8-37_ (P=0.008) but not AC187 (P=0.98). AC187, however, was able to block the above effect of AMY at the i.t. dose of 2.5 nmoles/10uL (P=0.00). The inhibitory effect of sCT on the c-Fos positive neurons in the dorsal spinal cord was antagonized, but not completely blocked upon pretreatment of rats by rAMY_8-37 _at either doses of 1.00 or 2.5 nmoles/10uL. The above-mentioned effect of sCT was not significantly reduced by 1.00 nmoles/10uL of AC187 but was reversed when the i.t. dose of the antagonist was raised to 2.5 nmoles/10uL (P=0.00). CGRP_8-37 _had no significant effect on the formalin-induced c-Fos expression. This antagonist was not also able to reverse the inhibitory effects of either AMY or sCT observed in this study. We also examined whether AMY-related antagonists can affect the expression of c-Fos when injected alone to control rats. As shown in figure 2, both AC187 and rAMY_8-37 _caused a moderate but significant bilateral increase of the Fos-like immunoreactivity in the lumbar spinal cord two hours after they were injected i.t. to control rats. CGRP_8-37 _had no significant effect in this regard (table 2).

**Table 2:** Effects of AMY antagonists on the c-Fos expression at the L3-L5 levels of the rat spinal cord

**Treatments (i.t.)**	**Total c-Fos Immunoreactive nuclei in laminae I-II and IV-VI **
Saline	3.00±0.57^a^
AC187 (1 nmoles/10uL)	10.66±0.88^b^
rAMY_8-37_ (1 nmoles/10uL)	12.66±1.76^b^
CGRP_8-37_ (2.5 nmoles/10uL)	4.33±1.71^a^

Data are Mean±SEM (n=3) of the number of c-Fos positive nuclei per section. Significant differences between groups were evaluated using one-way ANOVA (F (3, 8)=12.16, P=0.00) followed by Tukey post hoc analysis to assess significant differences between each group. The values carrying different letters are significantly different at P<0.05. Values with the same letters are not significantly different at P<0.05. Antagonist peptides and saline were injected i.t.

## Discussion

Nuclear visualization of Fos-like immunoreactivity that peaks 2 h after neurons are stimulated, is the best marker of neuronal activation.^33^ As such, we have already shown that i.t. injection of two peptides of the calcitonin family, CGRP and adrenomedullin, increase c-Fos expression in the spinal cord of rats. As shown by the present results, the two other members of this family, AMY and sCT, did not track CGRP and adrenomedullin and failed to induce the expression of c-Fos. Rat α-CGRP reportedly has agonistic effects at rat AMY 1(a) and rat AMY 3(a) receptors with potencies equivalent to rat AMY.^21^ The fact that rat α-CGRP and AMY show contradictory effects on the number of spinal c-Fos positive nuclei, implies that the nature of rat AMY receptors in the rat spinal cord may be different from the classical rat AMY receptors.

By using Western blotting protocol, Amylin is shown to inhibit c-Fos expression following visceral pain.^9^ Immunohistochemistry data have shown that a number of spinal neurons express c-Fos in response to formalin induced inflammation in the rat paw. These neurons are mainly located in laminae I and II, and laminae V and VI of the dorsal horn.^33^^,^^35^ Here, we have used Immunohistochemistry to show that both AMY and its structurally related peptide, sCT inhibit formalin induced c-Fos expression in neurons located in the above mentioned spinal laminae of rats. This finding implies that AMY and sCT may have antinociceptive properties against inflammatory pain. The two peptides should be tested in animal models of inflammatory pain to confirm this notion.

AMY binding sites were initially identified in the rat brain as CT-sensitive CGRP binding sites, but AMY receptors in the rat spinal cord are not characterized and their molecular components are not defined. Receptors for AMY are heterodimers of CTR and RAMPs.^37^ To our knowledge, mRNAs encoding the three RAMPs are expressed in the spinal cord, but the expression of CTR encoding mRNA is not clearly shown in this tissue.^17^^,^^18^ However, binding sites for sCT is shown in the spinal cord of rats.^18^ Whether sCT binding sites exactly mirror AMY sites of action in the spinal cord and whether these sites are presynaptic, postsynaptic or both are unknown.

Antinociceptive effects of Amylin on visceral pain are shown to be inhibited by the Amylin antagonist, Salmon calcitonin_8-32_.^11^ In the second part of this study, we used AMY receptor antagonists with different affinities for the classical AMY receptors in order to probe the nature of the receptors that mediate effects of AMY and sCT on the formalin induced c-Fos expression.

rAMY_8-37 _is generally considered a weak antagonist of rat AMY,^21^^,^^38^^,^^39^ whereas AC187 is considered as a selective and potent AMY antagonist.^24^ CGRP_8-37 _reportedly^28^^,^^29^ shows antagonistic effects at both CGRP and AMY receptors. When tested in cell culture studies, the order of potency of the antagonist peptides at transfected AMY_1(a)_ and AMY_ 3(a)_ receptors were AC187> rα-CGRP_8-37_>> rAMY_8-37_.^40^ However, the present data show that in the context of spinal c-Fos expression, effects of CGRP_8-37 _were not in line with those of the rAMY_8-37_ and/or AC187. rAMY_8-37 _blocked the effects of AMY on formalin induced c-Fos expression while CGRP_8-37 _failed to do so when injected at equimolar concentrations as rAMY_8-37_. This fact is in line with the conclusion that AMY may act through undefined receptors to inhibit the effects of intraplantar formalin on the expression of c-Fos in the rat spinal cord. Moreover, our data imply that the antagonist potency of rAMY_8-37 _at AMY receptors in the rat spinal cord may be as potent as, if not more potent than AC187. The reason is that AMY_8-37 _blocked the inhibitory effect of AMY on the formalin-induced c-Fos expression when administered at 20 times the concentration of the agonist. Whereas AC187 failed to show significant antagonistic effects at the above mentioned sites when administered at the same dose as AMY_8-37_. Thus, whether yet uncharacterized combinations of RAMPs and CTR splice variants or alternative receptors produce AMY and sCT receptors in the rat spinal cord, needs further investigation. Intrathecal administration of AC187 and or rAMY_8-37 _to rats injected with intraplantar saline lead in a remarkable increase in the number of Fos positive nuclei in the spinal cord. This effect can be attributed to the antagonistic action of AC187 and or rAMY_8-37 _at the action sites of an endogenous ligand (i.e. AMY) in the spinal cord. CGRP_8-37 _did not affect the expression of c-Fos under similar conditions as above.

The intracellular mechanism of the expression of the c-Fos gene induced by AMY and sCT in adult rat spinal cord remains to be clarified. Taken together, our results demonstrate that both AMY and sCT are inhibitory to neurons in the rat spinal cord and act via undefined receptors. 
